# Pharmacogenetic Interventions Improve the Clinical Outcome of Treatment-Resistant Autistic Spectrum Disorder Sufferers

**DOI:** 10.3390/pharmaceutics14050999

**Published:** 2022-05-06

**Authors:** Maria J. Arranz, Juliana Salazar, Valentin Bote, Alicia Artigas-Baleri, Alexandre Serra-LLovich, Emma Triviño, Jordi Roige, Carlos Lombardia, Martha Cancino, Marta Hernandez, Marc Cendros, Enric Duran-Tauleria, Natalia Maraver, Amaia Hervas

**Affiliations:** 1 Fundació Docència i Recerca Mútua Terrassa, 08221 Terrassa, Spain; alexserra@mutuaterrassa.cat (A.S.-L.); martahh1@blanquerna.url.edu (M.H.); mcendros@gmail.com (M.C.); 2 Centro de Investigación Biomédica en Red de Salud Mental (CIBERSAM), 28029 Madrid, Spain; 3 Translational Medical Oncology Laboratory, Institut de Recerca Biomèdica Sant Pau (IIB-Sant Pau), 08041 Barcelona, Spain; jsalazar@santpau.cat; 4 Department of Child Psychiatry, Hospital Universitari Mútua Terrassa, 08221 Terrassa, Spain; vbote@mutuaterrassa.cat (V.B.); mccancino@mutuaterrassa.cat (M.C.); 32989ahz@comb.cat (A.H.); 5 Genetics Department, Hospital de la Santa Creu i Sant Pau, 08025 Barcelona, Spain; a.artigasbaleri@gmail.com; 6 Genetics Department, Catlab, Viladecavalls, 08232 Barcelona, Spain; etrivino@catlab.cat (E.T.); jroige@catlab.cat (J.R.); clombardia@catlab.cat (C.L.); 7 School of Health Sciences Blanquerna, University Ramon Llull, 08024 Barcelona, Spain; 8 EUGENOMIC Genómica y Farmacogenética, 08029 Barcelona, Spain; 9 Institut Global d’Atenció Integral al Neurodesenvolupament (IGAIN), 08007 Barcelona, Spain; eduran@igain.cat (E.D.-T.); nmaraver@igain.cat (N.M.)

**Keywords:** ASD, pharmacogenetic intervention, pharmacotherapy, personalisation of treatment, antipsychotics, antidepressants, anxiolytics

## Abstract

BACKGROUND: Autistic spectrum disorders (ASD) are severe neurodevelopmental alterations characterised by deficits in social communication and repetitive and restricted behaviours. About a third of patients receive pharmacological treatment for comorbid symptoms. However, 30–50% do not respond adequately and/or present severe and long-lasting side effects. METHODS: Genetic variants in *CYP1A2*, *CYP2C19*, *CYP2D6* and *SLC6A4* were investigated in N = 42 ASD sufferers resistant to pharmacological treatment. Clinical recommendations based on their pharmacogenetic profiles were provided within 24–48 h of receiving a biological sample. RESULTS: A total of 39 participants (93%) improved after the pharmacogenetic intervention according to their CGI scores (difference in basal-final scores: 2.26, SD 1.55) and 37 participants (88%) according to their CGAS scores (average improvement of 20.29, SD 11.85). Twenty-three of them (55%) achieved symptom stability (CGI ≤ 3 and CGAS improvement ≥ 20 points), requiring less frequent visits to their clinicians and hospital stays. Furthermore, the clinical improvement was higher than that observed in a control group (N = 62) with no pharmacogenetic interventions, in which 66% responded to treatment (difference in CGI scores: −0.87, SD 9.4, *p* = 1 × 10^−5^; difference in CGAS scores: 6.59, SD 7.76, *p* = 5 × 10^−8^). CONCLUSIONS: The implementation of pharmacogenetic interventions has the potential to significantly improve the clinical outcomes in severe comorbid ASD populations with drug treatment resistance and poor prognosis.

## 1. Introduction

Autistic spectrum disorders (ASD) are severe neurodevelopmental alterations characterised by deficits in social communications and repetitive and restricted behaviours. The prevalence of ASD in the population is 0.8% with a male/female ratio of 3:1 [1]. Comorbidity is the principal cause of hospitalisation and poor prognosis. Chronic anxiety and depression are frequent symptoms in adolescents and adults with ASD, and suicide is also increased in this population. A high proportion of ASD people also have associated chronic aggression and self-harming behaviour. Although there is no specific pharmacological treatment for ASD, about a third of patients receive treatment for comorbid symptoms and 41% are prescribed more than one psychotropic drug [2]. Stimulant, anxiolytic, antipsychotic and antidepressant drugs are used for the treatment of conduct, anxiety and mood disorders observed in ASD patients. However, there is significant individual variability in the response to pharmacological treatment. Not all ASD patients respond to pharmacological treatment, with 30–50% not responding and/or presenting severe and long-lasting side effects [3,4]. Common side effects associated with the use of psychotropic treatments include increased irritability, aggressiveness and somnolence, movement disorders, weight gain and hormonal changes [5,6]. Negative cardiometabolic abnormalities are observed in 60% of ASD children treated with antipsychotics [7]. Other rarer but more severe side effects include neuroleptic malignant syndrome, hypersensitivity and suicidal ideation [5]. Furthermore, children and adolescents are more susceptible to drug-induced side effects than adults [8]. Antipsychotic medications with modest side effects in adult patients can cause significant weight gain in ASD children [7]. Conflicting data associated the use of antidepressant medications with higher rates of suicidal ideation in children and adolescents, although a recent meta-analysis disputes this finding [9]. Treatment discontinuation due to poor response and/or side effects has a negative effect on ASD patients’ prognosis, and predictors of response for the personalisation of pharmacological treatment are needed. 

There is strong evidence of the influence of genetic factors on the clinical outcome of pharmacological treatments. Several studies have associated genetic variants in the serotonin pathway and response to antidepressant medications [10,11]. Functional polymorphisms in hepatic cytochrome P450 (CYP) enzymes involved in the metabolism of more than 80% of existing drugs have been shown to influence drug plasma levels and bioavailability and to be associated with drug efficacy and induced side effects [12]. Furthermore, pharmacogenetic tests interrogating functional polymorphisms in key CYP enzymes (CYP1A2, CYP2C19 and CYP2D6) and antidepressant targets (serotonin transporter, 5-HTT and serotonin receptor type 2A, 5-HT2A) may help to improve clinical outcome by providing information to adjust doses and/or select treatments [13,14,15]. Pharmacogenetic-guided prescription of antidepressants also resulted in improved adherence and reduced pharmacy costs [16,17]. In a previous study, we showed that pharmacogenetic interventions may also help to improve the safety profile of antipsychotic medications [18]. However, only a modest number of pharmacogenetic studies have been conducted on ASD populations, and pharmacogenetic interventions have not been attempted for the personalisation of their treatment [19].

Despite the encouraging data, pharmacogenetic interventions are rarely implemented in clinical settings. Lack of knowledge of the clinical applicability, expensive and time-consuming tests and lack of clinical intervention criteria may hinder the implementation of pharmacogenetics as a prescription tool. Furthermore, the evidence of the clinical and economic benefits of implementing genetic tests for the improvement of pharmacotherapy is limited, particularly in children and adolescent populations. Most pharmacogenetic studies have been conducted on adult populations, and intervention tests have not been investigated in the ASD population. In this study, we introduced pharmacogenetic interventions to adjust clinical doses and select adequate drugs in ASD associated with comorbidity or coexistence with other disorders. Rapid and low-cost pharmacogenetic methods were developed to facilitate their implementation in clinical settings, and clinical recommendations criteria were standardised by child psychiatrists. We hypothesised that pharmacogenetic interventions may benefit the clinical outcome of psychotropic treatments in otherwise treatment-resistant patients. The highly positive results of this study indicate that pharmacogenetic interventions may be a helpful tool to significantly improve the efficacy and safety of pharmacotherapy in treatment-resistant ASD sufferers and to reduce health care costs.

## 2. Materials and Methods

### 2.1. Study Cohort

Table 1 summarises the demographic and clinical data of the study participants.

Treatment resistant: A total of 42 individuals (31 males and 11 females, average age: 18·70 ± 8·31 SD) with ASD diagnosis according to DSM-5 criteria and attending the ASD specialised unit at Hospital Universitari Mutua de Terrassa (HUMT, Terrassa, Barcelona, Spain) were included in this observational study. All of the patients were resistant to treatment and had undergone unsuccessful treatment with at least two drugs. The medications used included antipsychotics (risperidone (43%), aripiprazole (26%), olanzapine (14%), quetiapine (11%), paliperidone (6%)), antidepressants (fluoxetine (39%), sertraline (26%), paroxetine (13%), fluvoxamine (3%), escitalopram (3%), duloxetine (9%), desvenlafaxine (6%)) and anxiolytics or antiepileptics (benzodiazepines (31%), lithium (21%), methylphenidate (14%), guanfacine (14%), valproate (10%), levomepromazine (7%), clonidine (3%)). Three participants were not receiving medication at the time of the intervention, having previously failed to three or more medications. Thirteen participants were on monotherapy with one medication, and the rest (N = 26) were treated with a combination of drugs (see Table 1). Patients were referred to our pharmacogenetics team for testing of key genetic markers and a recommendation report. Control group: A total of 62 individuals (57 males and 5 females, average age: 13.83 ± 3.8) with ASD diagnosis according to DSM-5 criteria were included in the study. No pharmacogenetic intervention was performed in the control group, which included 21 patients who did not respond to pharmacological treatment (no improvement in either CGI or CGAs scores) and 41 patients who showed treatment response (improvement in both CGI and CGA scores). All the participants or their carers gave informed consent to participate in the study. The study was approved by the HUMT Ethics Committee. 

### 2.2. Pharmacogenetic Intervention

A saliva or blood sample was obtained from the treatment-resistant patients and sent to our pharmacogenetics laboratory for testing. DNA samples were extracted using commercial kits: SQ Blood and Saliva DNA kits (Omega Bio-Tek, Norcross, GA, USA), and following the manufacturer’s directions. Key pharmacogenetic polymorphisms in metabolic enzymes and drug targets were genotyped (see Table 2). Rapid methods for the characterisation of these polymorphisms were standardised using Taqman probes (Applied Biosystems, Foster City, CA, USA) for the detection of SNPs and CNVs in *CYP1A2*, *CYP2C19* and *CYP2D6* genes, and the PCR and RFLP methods for the detection of variants in the gene coding for the serotonin receptor protein, *SLC6A4.* QuantStudio 3 (Applied Biosystems) and QIAxcel Advanced System (Qiagen, Hilden, Germany) technologies were used for the genotyping. Detailed information on the genotyping protocols used can be provided on request. Functional phenotypes of CYP enzymes (normal, intermediate, poor, rapid or ultra-rapid metabolisers) were determined following the Clinical Pharmacogenetics Implementation Consortium (CPIC) guidelines (www.cpicpgx.org, accessed on 1 January 2018). Information on 22 drugs used in the treatment of ASD comorbidities and for which pharmacogenetic guidelines existed was provided (see Table 3). Drugs were classified as not affected, moderately affected and significantly affected by the genetic variants observed in their metabolic enzymes and/or drug targets. Clinical recommendations were provided based on CPIC and Dutch Pharmacogenetic Working Group (DPWG, www.knmp.nl, accessed on 1 January 2018) guidelines and our own results [18], as well as based on the information published for *SLC6A4* [10]. A report including the pharmacogenetic profile and the clinical recommendations was issued to the participants’ clinicians within 24–48 h of receiving the blood or saliva sample in our laboratory. Upon receiving the report, the clinicians decided if a dose adjustment or a change to drugs, which were predicted to have a higher efficacy and/or safety, was required. ASD treatment-resistant patients with functional variants in the enzyme CYP2D6 were treated with alternative drugs not metabolised by the enzyme (i.e., sertraline, citalopram, clozapine or quetiapine). Patients with functional variants in the enzyme CYP2C19 were treated with drugs mainly metabolised by other enzymes (i.e., fluoxetine, paroxetine, clozapine or quetiapine). Patients with ultra-rapid variants of CYP1A2 were treated with higher doses of clozapine or given alternative treatment not metabolised by this enzyme (i.e., aripiprazole, risperidone, quetiapine or paliperidone). Patients with the *SLC6A4* LPR s/s genotype associated with reduced expression of the serotonin transporter (5-HTT), the main target of selective serotonin reuptake inhibitors (SSRIs), were given alternative treatments (atomoxetine, guanfacine or antipsychotics such as clozapine, olanzapine and risperidone). When several genetic contraindications were observed in the same individual, the drugs that required less adjustments were chosen. 

### 2.3. Clinical Assessment of Response

**Figure 1:**
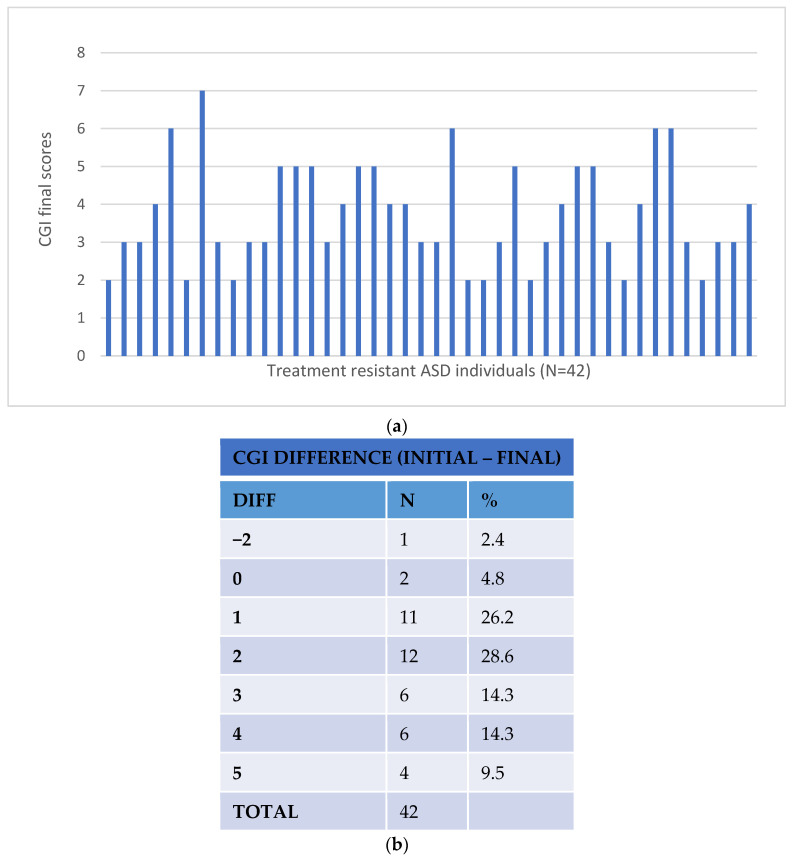
CGI results after pharmacogenetic intervention: (**a**) final CGI scores; (**b**) CGI score difference (CGI basal–final).

**Figure 2:**
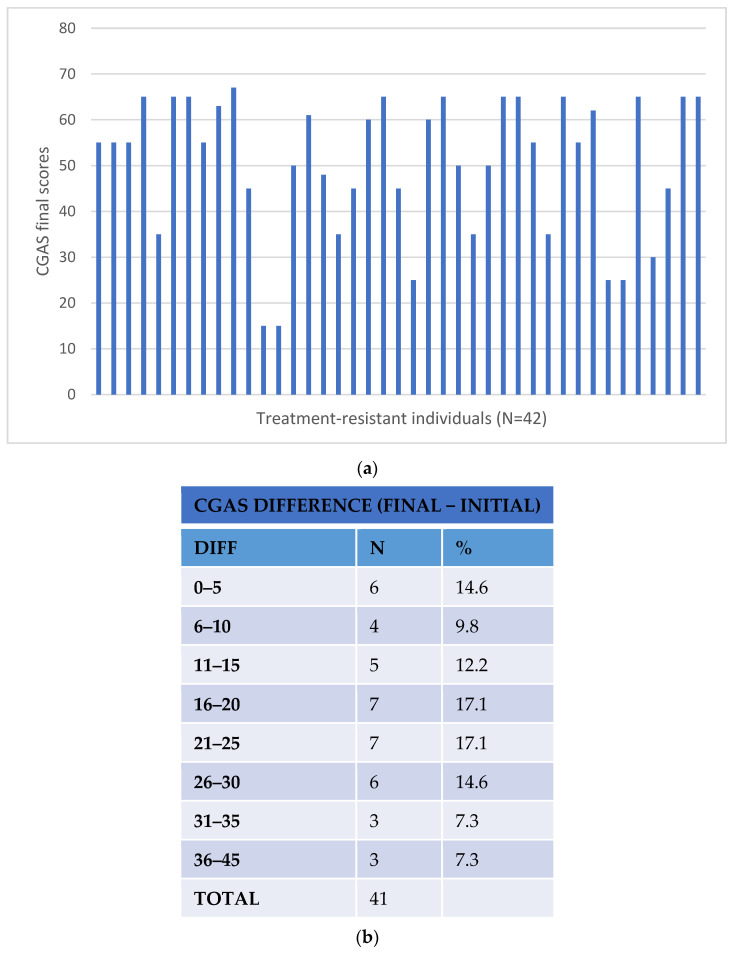
CGAS results after pharmacogenetic intervention: (**a**) final CGI scores; (**b**) CGAS score difference (CGAS final–basal).

Response to treatment was assessed using the Clinical Global Impression (CGI) and the Children’s Global Assessment Scales (CGAS) [20,21]. The level of response was determined by the differences in the CGI and CGAS scales before the pharmacogenetic intervention (basal) and after at least eight weeks of the resulting intervention (clinical dose adjustment or treatment change). Improvements of 3 or more in the CGI scores and of 20 or more in the CGAS scores were considered significant as the patient reached stabilisation and required less medical attention (no hospitalisation, less frequent visits to their clinician and no change or additional medication) [22]. Figure 1 and Figure 2 represent the changes in the CGI and CGAS scores pre- and post-pharmacogenetic intervention in the treatment-resistant cohort patients. The response assessments of all the study participants, treatment-resistant and control subjects, were performed by their own clinician.

### 2.4. Statistical Analyses

Linear regression analysis considering the difference in CGI or CGAS scores before and after treatment as the dependant variables, age and gender as covariates, and pharmacogenetic intervention (yes/no) as predictor variable were performed on the total cohort (N = 104). The analysis model was repeated including patients with pharmacogenetic-guided treatment and control individuals that did not show response to explore the effect on the intervention on treatment-resistant patients. A final analysis including patients with pharmacogenetic interventions and control individuals who responded to their initial treatment was also performed to further asses the efficacy of the intervention. All the analyses were performed using the SPSS statistical package (IBM, version 28, Armonk, NY, USA).

## 3. Results

Genetic variants predicting altered metabolising rates of the drugs investigated and/or reduced expression or altered activity of the 5-HTT target protein were observed in all treatment-resistant patients. Twenty four percent of the resistant ASD subjects presented CYP genetic variants predicting an altered metabolism; five percent presented genetic alterations in the drug targets associated with poor efficacy and safety; and the rest (71%) presented genetic variants predicting poor response or side effects in both CYP enzymes and drug targets. 

The frequency of predicted CYP1A2 ultra-rapid metabolisers (44%, see Table 4) in this group was higher than that observed in our previous study in an adult population of the same geographical and ethnic origin (36%) [18]. The frequency of genetically determined CYP2C19 ultra-rapid metabolisers was also higher than in our previous study (34% vs. 25%, respectively). A high prevalence of genetically predicted CYP2D6 poor and ultra-rapid metabolisers (7% in both cases) in comparison with other Spanish populations was also observed [23]. However, these values were within the ranges observed in other European populations [12]. The frequency of genetic variants associated with a reduced expression of the 5-HTT protein (29%) was also higher than the average observed in healthy Spanish individuals and bipolar patients (24% and 22%, respectively) [24]. 

A total of 39 treatment-resistant patients (93%) showed improvement after changes to their treatment based on their pharmacogenetic results according to the CGI scores with a difference (CGI basal–final scores) of 2.26 (SD 1.55) (see Figure 1). According to the CGAS values, 37 participants (88%) showed improvement after the pharmacogenetic intervention (decrease in CGA scores ≥ 20), with an average improvement (final–basal CGA scores) of 20.29 (SD 11·85 (see Figure 2)). These figures were an improvement on those observed in the control group, where the difference in CGI scores observed after treatment was −0.87 ± 9.44 and the difference in CGA scores was 6.59 ± 7.75. The regression analyses revealed these differences to be statistically significant (likelihood ratio χ^2^ = 25.62, *p* = 1 × 10^−5^ for CGI scores and χ^2^ = 36.72, *p* = 5 × 10^−8^ for CGA scores, considering age and gender as covariates). The superior improvement in the treatment-resistant group after pharmacogenetic intervention was more evident when comparing with the non-responders (no improvement in CGI or CGA scores) in the control group (likelihood ratio χ^2^ = 31.74, *p* = 5 × 10^−7^ for CGI scores and χ^2^ = 47.09, *p* = 3 × 10^−10^ for CGA scores, considering age and gender as covariates). It is of note that the improvement in the pharmacogenetic-guided group was marginally higher than that observed in the control patients who responded to treatment (likelihood ratio χ^2^ = 8.37, *p* = 0.04 for CGI scores and χ^2^ = 11.45, *p* = 0.01 for CGA scores, considering age and gender as covariates, see Table 5). 

Finally, according to their CGI and CGAS values, 23 of the treatment-resistant patients (55%) were considered to have achieved symptom stability (CGI ≤ 3 and CGAS improvement ≥ 20 points), requiring less frequent visits to their clinicians (a reduction of 10 visits/patient/year: 230 less visits per year) and a reduction in hospital stays (approximately a total reduction of three months in hospital stays).

## 4. Discussion

There is great variability in the responses to pharmacotherapy in ASD subjects. Genetic variants in metabolic enzymes and drug targets may influence response to medication in these patients. This is an especially important issue considering the high rate of chronic anxiety, depression, aggression and suicide associated with ASD. Pharmacogenetic-guided adjustments may help improve the response of otherwise treatment-resistant patients. In this study, we used pharmacogenetic tests to guide treatment in treatment-resistant ASD subjects and observed significant improvements after adjusting clinical doses or selecting alternative treatments according to the individuals’ pharmacogenetic profile. 

All of the ASD treatment-resistant subjects in the study showed genetic variants associated with poor response and/or adverse reactions to the treatments they had received or were receiving, which, in addition to clinical and environmental factors, may have significantly contributed to treatment failure. Ninety five percent of them presented functional variants in the *CYP1A2*, *CYP2C19* or *CYP2D6* genes, which predicted alterations in their metabolic rates associated with increased drug toxicity and inefficacy. The prevalence of these *CYP* functional variants in the treatment-resistant cohort was higher than that observed in other Spanish cohorts [18,23], although they were within the ranges described for Caucasian individuals [12]. An excess of *CYP2D6* functional variants has previously been reported in children resistant to treatment with psychotropics [25] and in ASD subjects [26]. *CYP2D6* variants were also reported to be associated with risperidone-induced side effects in ASD children [27]. CYP2C19 ultra-rapid metabolisers were associated with reduced tolerance of the selective serotonin reuptake inhibitor (SSRI) escitalopram in ASD subjects [28]. *CYP* functional variants were also observed in an adolescent who did not tolerate pharmacotherapy [29]. An excess of *CYP2C9* risk variants was also observed in ASD patients with gastrointestinal dysfunction [30]. In summary, these findings support a significant role of pharmacokinetic genetic variants in determining treatment response and highlight the importance of the genotyping of CYP functional variants to adjust clinical doses accordingly. Functional variants in CYP enzymes are known to affect the plasma levels of drugs, and their metabolites and are directly associated with the clinical outcome of pharmacotherapy [10]. Poor metabolisers of CYP tend to display more side effects than normal or in comparison to ultra-rapid metabolisers, whereas ultra-rapid metabolisers of CYP usually present suboptimal responses. Although in our study we did not assess treatment induced side effects, it is likely that a pharmacogenetic intervention adjusting the clinical doses according to the CYP profile of the patients will significantly reduce adverse reactions in ASD subjects and merits further investigation. Although CYP metabolic rates may also be influenced by environmental (i.e., diet and smoking habits) and clinical (i.e., concomitant treatment) factors, knowledge of the genetically determined alterations may help in selecting the right dose for each patient and significantly increase drug safety and efficacy.

Regarding drug targets, a high frequency of *SLC6A4* genetic variants associated with poor efficacy and/or increased frequency of side effects was also observed. The frequency of genetic variants associated with reduced expression of the 5-HTT protein was also higher in treatment-resistant ASD subjects than the average observed in healthy Spanish individuals [24]. This is not surprising given that most treatment-resistant participants (around 60%) had been or were being unsuccessfully treated with SSRI antidepressants, a first-line treatment option for depression in ASD patients. SSRIs’ mechanism of action includes the blocking of the 5-HTT protein to inhibit the reuptake of serotonin. Several studies have shown that presence of *SLC6A4* LPR short variants and STin2 12 repeat alleles are associated with a poorer response to SSRI antidepressants in Caucasian populations [10,31]. Greater irritability according to the Aberrant Behaviour Checklist (ABC) subscale was also observed in ASD children treated with citalopram and presenting the *SLC6A4* LPR s/s genotype [32]. Thus, the high prevalence of *SLC6A4* risk variants in a treatment-resistant ASD cohort agrees with previous findings. Our pharmacogenetic report included a recommendation to use alternatives to SSRI antidepressants if the individual presented *SLC6A4* risk variants.

The adjustment of doses according to the functional variants observed in CYP genes and the selection of alternatives when *SLC6A4* risk variants were observed resulted in the improvement of drug efficacy and safety in at least 88% of the treatment-resistant ASD subjects. The clinical improvement after the pharmacogenetic intervention was superior to that observed in the control group without pharmacogenetic intervention, which included 66% of patients classified as responders (improvement in CGI and CGA scores after treatment). Furthermore, 23 of the treatment-resistant ASD subjects (54%) achieved clinical stability after the pharmacogenetic intervention, requiring a smaller number of visits to their clinicians. Additionally, a total reduction in hospital stays of approximately three months was also achieved. It is of note that clinicians were not able to follow the pharmacogenetic recommendations in two of the subjects who did not show any improvement due to patient unwillingness to change or initiate a new treatment. Taken together, these results indicate that the pharmacogenetic intervention resulted in significant clinical and economic benefits.

Our study has several limitations. First, the sample size was moderate, although sufficient to observe the effect of the pharmacogenetic intervention. Second, only the most frequent variants observed in the Spanish population (>99% of variation) were investigated so as to reduce the price and time required to deliver results. The control group was younger and less severely affected than the treatment-resistant cohort, as the latter included patients who had been resistant to treatment for several years. The pharmacogenetic report provided to the clinician was based on genetically predicted metabolic rates and expression. However, the possible effect of concomitant treatment and other phenoconversion factors was not considered, although a list of possible inducers and inhibitors of the enzymes investigated was provided in the report. Finally, compliance could not be confirmed via plasma levels, although treatment adherence was supervised by parents and carers.

In summary, these results reflect the contribution of genetic factors to pharmacotherapy resistance in ASD subjects and highlights the importance of conducting pharmacogenetic testing for the personalisation of their treatments. The implementation of pharmacogenetic interventions has the potential to significantly improve clinical outcomes in severe comorbid ASD populations with drug treatment resistance and poor prognosis.

## Figures and Tables

**Table 1:** Clinical and demographic data of investigated cohorts.

**Clinical and Demographic Data**		
**Total N = 104**		
Treatment-resistant cohort	N	42
	age	18·79 ± 8.3 SD ^3^
	Gender	74% male ^4^
	Basal CGI	6 ± 0.99
	Basal CGAS	30.02 ± 13.28
Control	N	62
	age	13.83 ± 3.8
	Gender	92% male
	Basal CGI	4.33 ± 0.80
	Basal CGAS	44.32 ± 9
**Medication**		
	**Treatment resistant**	**Control**
Antipsychotics ^1^	28 (67%)	29 (32%)
Antidepressants ^2^	20 (48%)	10 (11%)
Anxiolytics, anticonvulsants and others	21 (26%)	50 (56%)
No current medication	3 (7%)	0

^1^ Antipsychotic drugs used: risperidone (43%), aripiprazole (26%), olanzapine (14%), quetiapine (11%), paliperidone (6%). ^2^ Antidepressant drugs used: fluoxetine (39%), sertraline (26%), paroxetine (13%), fluvoxamine (3%), escitalopram (3%), duloxetine (9%), desvenlafaxine (6%). ^3^ The treatment-resistant cohort was relatively older than the control group (*p* = 0.001). ^4^ Gender distribution was not significantly different between cohorts (*p* = 0.10).

**Table 2:** Key pharmacogenetic variants investigated.

Gene	Variants Studied	
*CYP1A2*	rs762551 (*1F)	A > C
*CYP2C19*	rs4244285 (*2)	G > A
	rs12248560 (*17)	C > T
*CYP2D6*	rs35742686 (*3)	delA
	rs3892097 (*4)	G > A
	rs5030655 (*6)	delT
	rs5030656 (*9)	delAAG
	rs1065852 (*10)	C > T
	rs28371706 (*17)	C > T
	rs28371725 (*41)	G > A
	Gene deletion (*5)	-
	Gene duplication (*XN)	-
*SLC6A4*	LPR	L/S
	rs25331	A > G
	STin2	12/10/9

LPR: linked promoter region; STin2: serotonin transporter intron 2.

**Table 3:** List of drugs and the relevant metabolic enzymes and targets investigated.

Drug	Type	CYP Enzymes	Targets
Amitriptiline	TCA	CYP2C19, CYP2D6	5HTT, 5-HT2A
Aripiprazole	SGA	CYP2D6	
Atomoxetine	NRI	CYP2D6	
Citalopram	SSRI	CYP2C19	5-HTT
Clomipramine	TCA	CYP2C19, CYP2D6	5-HTT, 5-HT2A
Clozapine	SGA	CYP1A2, CYP2C19	
Desvenlafaxine	SNRI		5-HTT
Duloxetine	SNRI	CYP2D6 & CYP1A2	5-HTT
Escitalopram	SSRI	CYP2C19,	5-HTT
Fluoxetine	SSRI	CYP2D6	5-HTT
Fluvoxamine	SSRI	CYP2D6	5-HTT
Haloperidol	FGA	CYP2D6	
Imipramine	TCA	CYP2C19, CYP2D6	5-HTT
Maprotiline	TCA	CYP2D6	
Mirtazapine	TCA	CYP2D6, CYP1A2	5-HT2A
Nortriptyline	TCA	CYP2D6	5-HTT, 5-HT2A
Olanzapine	SGA	CYP1A2	
Paroxetine	SSRI	CYP2D6	5-HTT
Risperidone	SGA	CYP2D6	
Sertraline	SSRI	CYP2C19	5-HTT
Venlafaxine	SNRI	CYP2D6	5-HTT
Vortioxetine	SMS	CYP2D6	5-HTT

Abbreviations: FGA: first-generation antipsychotics, SGA: second-generation antipsychotic, SNRI: serotonin-norepinephrine reuptake inhibitor, SSRI: selective serotonin reuptake inhibitor, SMS: serotonin modulator and stimulator, TCA: tricyclic antidepressant.

**Table 4:** Summary of predicted phenotypes observed in treatment-resistant patients.

**Gene**	**CYP1A2**	**CYP2C19**	**CYP2D6**
**Phenotype**			
Normal metaboliser	56%	34%	81%
Intermediate metaboliser	-	32%	5%
Poor metaboliser	-	-	7%
Rapid or ultra-rapid metaboliser	44%	34%	7%
**Gene**	**SLC6A4**		
**Phenotype**			
Normal expression or activity	20%		
Intermediate expression or activity	51%		
Reduced expression or activity	29%		

**Table 5:** Differences in CGI and CGA scores in the treatment-resistant and control groups observed after 12 weeks of treatment.

**Treatment-Resistant ASD Subjects (N = 42)** **(with Pharmacogenetic Intervention)**	
Improvement in CGI scores (basal–final scores)	2.26 ± 1.55
Improvement in CGA scores (final–basal scores)	20.29 ± 11.85
**Control subjects (N = 62)**	
Improvement in CGI scores (basal–final scores)	−0.87 ± 9.44
Improvement in CGA scores (final–basal scores)	6.59 ± 7.76
**Non-Responders (N = 21)** **(no improvement in CGI or CGA scores)**	
Improvement in CGI scores (basal–final scores)	0.10 ± 0.63
Improvement in CGA scores (final–basal scores)	0.48 ± 2.18
**Responders (N = 41)** **(Improvement in CGI and CGA scores)**	
Improvement in CGI scores (basal-final scores)	1.54 ± 0.588
Improvement in CGA scores (final-basal scores)	11.74 ± 7.17

## Data Availability

The data presented in this study are available on request from the corresponding author. The data are not publicly available due to privacy protection restrictions.
